# Vitamin D intake in mid-pregnancy and child allergic disease – a prospective study in 44,825 Danish mother-child pairs

**DOI:** 10.1186/1471-2393-13-199

**Published:** 2013-10-31

**Authors:** Ekaterina Maslova, Susanne Hansen, Camilla B Jensen, Andrew L Thorne-Lyman, Marin Strøm, Sjurdur F Olsen

**Affiliations:** 1 Centre for Fetal Programming, Department of Epidemiology Research, Statens Serum Institut, Artillerivej 5, 2300 Copenhagen, Denmark; 2 Institute of Preventive Medicine, Bispebjerg and Frederiksberg Hospital, Capital Region, Nordre Fasanvej 57, 2000 Frederiksberg, Denmark; 3 The Earth Institute, Columbia University, 535 West 116th Street, New York, NY 10027 USA; 4 Department of Nutrition, Harvard School of Public Health, 677 Huntington Ave, Boston, MA 02115 USA

**Keywords:** Cohort, Pregnancy, Vitamin D, Asthma, Allergic rhinitis

## Abstract

**Background:**

Past studies suggest that maternal vitamin D intake during pregnancy may protect against child wheeze but studies on asthma are limited. Our objective was to examine the relation between intake of vitamin D in mid-pregnancy and child asthma and allergic rhinitis at 18 months and 7 years.

**Methods:**

We examined data from 44,825 women enrolled during pregnancy in the longitudinal Danish National Birth Cohort (1996–2002). We estimated vitamin D intake from diet and supplements based on information from a validated food frequency questionnaire completed in gestational week 25. At 18 months, we evaluated child asthma using data from phone interviews. We assessed asthma and allergic rhinitis by self-report at age 7 and asthma by using records from national registries. Current asthma at age 7 was defined as lifetime asthma diagnosis and wheeze in the past 12 months. We calculated multivariable risk ratios with 95% CIs comparing highest vs. lowest quintile of vitamin D intake in relation to child allergic disease outcomes.

**Results:**

The median (5%-95%ile) intake of total vitamin D was 11.7(3.0-19.4) μg/day (68% from supplements). In multivariable analysis, mothers in the highest (vs. lowest) quintile of total vitamin D intake were less likely to have children classified with current asthma at 7 years (Q5 vs. Q1: 0.74, 95% CI: 0.56, 0.96, P = 0.02) and they were less likely to have children admitted to the hospital due to asthma (Q5 vs. Q1: 0.80, 95% CI: 0.64, 1.00, P = 0.05). We found no associations with child asthma at 18 months or with allergic rhinitis at 7 years.

**Conclusions:**

Our findings suggest a weak inverse relationship between high total vitamin D and asthma outcomes in later, but not early, childhood. The data did not suggest a clear threshold of vitamin D intake above which risk of asthma was reduced.

## Background

The past decades have seen a rise in allergic diseases and it has been hypothesized that the increase in prevalence may have its roots *in utero*[1-3]. Recently, low maternal levels of vitamin D have moved to the forefront as a potential causal agent [4]. Vitamin D is a prehormone that can be synthesized in the skin from 7-dehydrocholesterol when exposed to UV light. Through a series of hydroxylation steps vitamin D3 is converted to 25-hydroxyvitamin D (25(OH)D), the primary metabolite, and then 1,25-dihydroxyvitamin D (1,25(OH)_2_D), the active compound. Vitamin D can also be attained through diet from foods such as oily fish, eggs, fortified foods, and through the use of supplements [5]. In Denmark, no foods have, however, been enriched with vitamin D since 1985 [6]. The availability of few food sources, dietary changes away from fatty fish consumption, and the use of sunscreen, covering clothes and host factors (age, skin color, and body weight) have made vitamin D deficiency a ubiquitous problem [7-9]. In Denmark vitamin D deficiency (<20 nmol/L) was observed in 9% of ethnic Danish non-pregnant women [10]; 10% of women in a random sample from the cohort for the present study had 25(OH)D levels < 25 nmol/L [11]. While the importance of vitamin D for skeletal health has long been acknowledged, more recent discovery of receptors for vitamin D in multiple organ systems in the body, including immune cells, suggests that the health benefits of vitamin D may extend beyond its classic effects [12]. Vitamin D has also been shown to influence immunity by inducing cathelicidin, an antimicrobial peptide; activating T helper (Th) 1 and 2 cells through T regulatory (Treg) cells; and has been found in activated mononuclear leukocytes and lymphocytes [13-17].

In pregnancy, maternal 25(OH)D is the primary fetal vitamin D source, with some potential contribution from placental 1,25(OH)_2_D [18]. Epidemiological studies examining the intake of vitamin D during pregnancy have found inverse associations primarily with wheeze and eczema [19-21]; a recent study from the UK found no association with dietary vitamin D and wheeze, asthma, or sensitization [22]. Using either maternal or cord blood 25(OH)D as biomarker for fetal vitamin D exposure, studies have found mixed results of inverse [23-25], direct [26], and null [22,27] associations with respiratory and allergic disease outcomes. With few studies distinguishing between dietary and supplement sources of vitamin D and with only two studies including asthma [22,28] and allergic rhinitis [28] in children above 5 years of age, we decided to examine these relations in a large, prospective study. These results would be important not only for informing on recommendations for vitamin D intake during pregnancy in relation to asthma prevention, but also for determining the relevance of intake sources.

The objective of this study was to examine the association between vitamin D intake from diet and supplements during pregnancy and child allergic disease outcomes in early and later childhood using a large, longitudinal Danish pre-birth cohort.

## Methods

### Study population

The Danish National Birth Cohort (DNBC) is a large, prospective cohort study that enrolled women from 1996–2002 during their first antenatal visit (gestational week 6–10). Women were eligible if they could speak Danish and planned to carry to term. A total of 35% of all pregnancies in Denmark were enrolled (n > 100,000). Women partook in two interviews during pregnancy (gestational week 12 and 30) and two after birth (at 6 and 18 months). At the 25^th^ week of gestation they also filled out a validated, semi-quantitative food frequency questionnaire (FFQ) [29]. Mothers and children have been followed up through national registries and a questionnaire that was sent to the mothers when the child turned 7 years. Of the 101,045 enrolled pregnancies we included only singletons and first enrolled pregnancies to avoid dependencies among correlated measures (n = 87,056). Among the 61,546 women who had data on vitamin D intake from the FFQ, 44,825 had information on total (diet + supplement) vitamin D intake, limited by information on supplement use (n = 45,004). Women leaving the question on supplements blank accounted for the missing supplement data. Sample size further varied depending on missing outcome data.

Mothers provided written informed consent on behalf of their children. The Regional Scientific Ethics Committee for the municipalities of Copenhagen and Frederiksberg approved all study protocols, and all procedures were in accordance with the Declaration of Helsinki.

### Exposure assessment

Vitamin D intake was assessed using the mid-pregnancy-FFQ. Maternal intakes were estimated as described previously [30]. Briefly, specific questions were asked about intakes from approximately 360 different foods, and women were asked to report the frequency with which each had been consumed during the previous four weeks. We computed vitamin D intake by multiplying daily frequencies with standardized portions and calculated into micrograms per day using Danish food tables. All intakes were energy-adjusted using the residual method [31]. Women who reported intake resulting in unrealistic energy intake estimates (arbitrarily set to < 2500 kJ/day or > 25,000 kJ/day) were excluded (356/70,188(0.5%)). Intake from supplements were calculated from the information women provided on name of the product, the producer, nutrient content per daily dose, and number of daily doses. We compiled a data base of supplements using information from the Danish Veterinary and Food Administration. This information was combined with the frequency of intake reported by the women and daily intake based on daily recommended intake stated on the supplement. Majority of vitamin D supplements were in the form of D3, though a small proportion also contained D2. The data base is yet to be validated, but a similar data base has been validated in a Norwegian pre-birth cohort and showed good correlation with maternal 25(OH)D concentrations [32]. Furthermore, in our cohort we found that dietary and supplement vitamin D predicted maternal plasma 25(OH)D levels [11]. In our data the Spearman correlations for vitamin D from diet and supplements with plasma 25(OH)D were 0.07 (P = 0.05) and 0.26 (P < 0.001), respectively.

### Outcome assessment

We assessed outcomes at the 18 months and 7 year follow-up. *Asthma at 18 months* was defined as self-reported doctor asthma diagnosis. At the 7 year follow-up asthma and allergic rhinitis was assessed using the standardized International Study of Asthma and Allergies in Childhood (ISAAC) questionnaire which was mailed to the mothers [33]. *Current asthma at age 7* was defined using a combination of two questions including one on lifetime doctor diagnosis of asthma and one on wheezing symptoms in the past 12 months. Only children with positive answers to both of these questions were defined as having asthma at age 7. Children with positive answers to only one or to none of the questions were classified as not having asthma at age 7. Cases of *ever allergic rhinitis* were defined based on reported lifetime doctor diagnosis of hay fever.

We also used the mandatory Danish National Patient Registry (DNPR) that collects data on hospital admissions to define *ever admitted asthma*. The registry has demonstrated good validity against asthma diagnosis from hospital records [34]. We extracted data from the DNPR in August 2010 and linked it to our data using the unique identification number provided to all Danish citizens. Cases of *ever admitted asthma* were identified using the International Classification of Disease 10 (ICD-10) diagnosis codes for asthma (J45, J45.0, J45.1, J45.8, J45.9, J46, and J46.9). We included all first cases of admitted asthma over the entire study period (birth to 7 years of age).

The Register of Medicinal Product Statistics (RMPS) contains detailed individual-level dispensary information [35]. We used a previous validation study [36] to identify Anatomical Therapeutic Chemical codes for the classification of *ever prescribed asthma* cases. We defined a case as > =2 prescriptions for beta2agonists or steroids or > =1 prescription for any other obstructive airway disease drug. We included all first cases of prescribed asthma over the entire study period (birth to 7 years of age).

### Covariates

We collected and evaluated information on maternal sociodemographic and lifestyle covariates during pregnancy from the interviews including socioeconomic status by parental education level and occupation (SES: high level proficiency, medium level proficiency, skilled, unskilled, student, unemployed), maternal age at birth of child (<20, 20– < 25, 25– < 30, 30– < 35, 35– < 40, ≥40), parity (nulli- and multiparous), maternal prepregnancy BMI (in kg/m^2^) (<=18.5, 18.6-24.9, 25.0-29.9, 30–34.9, > = 35.0), maternal smoking (nonsmoker, occasional smoker, current smoker), maternal exposure to secondary smoke (yes/no), partner smoking (yes/no), maternal exercise (min/week), maternal outdoor activity (min/week), month and season of last menstrual period (LMP), maternal solarium used (weeks), gestational weight gain (g/week in quartiles), breastfeeding duration (none, 0–1, 2–3, 4–6, 7–9, > = 10 months), birth weight (in grams), gestational age (days since the LMP in quintiles), child sex, parental history of asthma and allergies. Dietary covariates which have previously shown associations with exposure or outcome were included into the multivariable model: energy, fruit and vegetables, alcohol consumption, and intake of n-3 fatty acids, vitamins A, C, E, folate, and calcium, selenium, and zinc from diet and supplements (reviewed in [37]). All covariates were self-reported except for maternal age, month of LMP, birth weight, and gestational age, which were extracted from the national registries. The percentage of missing values was low (0-1%) for majority of variables, apart from breastfeeding (27%) and gestational weight gain (32%). These covariates were poorly correlated with the exposure (Spearman rho < =0.01) and were deemed to be weak confounders. We therefore used the missing indicator method to account for the missing data. A complete-case analysis did not substantially alter the effect estimates though confidence intervals were slightly wider.

### Statistical analyses

We compared the distributions of vitamin D intake across categories of age-standardized covariates. Distributions were age-standardized due to significant differences in vitamin D intake across age categories with older women consuming more vitamin D compared to younger women. Intake of vitamin D was quintilized and indicator variables modelled comparing each quintile to the lowest, reference, quintile. We also examined potential continuous relations and dichotomized the exposure according to US (15 μg/d) [38] and Nordic (10 μg/d) [39] dietary recommendations. We further examined the possible non-linear relationship between both the exposures and covariates and odds ratio of child asthma non-parametrically with restricted cubic splines [40]. Tests for non-linearity used the likelihood ratio test, comparing the model with only the linear term to the model with the linear and the cubic spline terms. We found a non-linear relationship between season of LMP and birth weight with child asthma only. To account for the non-linearity we entered a cubic term into the final model.

In the multivariable models we introduced covariates that were associated with the outcome at P < 0.10 in univariate analyses. Covariates that were associated with the outcome at P < 0.05 in a multivariable model (excluding exposure), or for which there were biological priors (parental history of asthma/allergy and child sex) were included in the final models. Covariates initially suspected to be on the causal pathways, such as gestational weight gain, birth weight, and gestational age, were initially excluded from the model to avoid over-adjustment. Further adjustment for these covariates in the final model did not alter the results. Log-binomial models were used to estimate relative risks (RR) and 95% CI [41,42]. In a few instances, the models did not converge and log-Poisson models, which provide consistent but not fully efficient estimates of the RR and its confidence intervals, were used [43]. Median values for each quintile of exposure were entered separately into the models as a continuous variable to evaluate P-value for trend [44]. Interactions by parental asthma and allergy history, maternal vitamin A intake, and maternal prepregnancy BMI were examined using tertiles of vitamin D intake and evaluated using a robust score test. Variables were chosen on the basis of biological and physiological relevance to either the exposure (vitamin A, maternal prepregnancy BMI) or outcome (parental history of asthma and allergy). Statistically significant interactions were stratified upon.

All tests were two-sided and we used a threshold of p < 0.05 to denote statistical significance. The analyses were performed using the Statistical Analyses System software (release 9.3; SAS Institute, Cary, NC).

## Results

### Study population

A total of 44,825 mothers had information on total vitamin D intake. The majority of mothers were 25–35 years old (73%), of medium to high level proficiency (56%), and nulliparous (56%). Over 68% had a BMI in the normal range (18.5-24.9 kg/m^2^) and a mean gestational weight gain of 471 ± 212 g/week. A total of 12% reported daily smoking during pregnancy and the far majority of women did not exercise while pregnant (61%) or spend time doing outdoor physical activities (83%). Most mothers did not use tanning beds during pregnancy (74%). The LMP was evenly distributed among seasons with slightly more mothers reporting a LMP in the fall (26%) and winter (26%). Mean birth weight was 3,578 ± 571 g and mean gestational age 280 ± 14 days with 51% male births.

The median intake (5%-95%ile) of total vitamin D in the study population was 11.7(3.0-19.4) μg/day (3.0(1.5-7.4) μg/day from diet; 8.9(0.0-15.0) μg/day from supplements). While the range went all the way to 81.1 μg/day, this was mostly due to outliers. Nine percent (4,260/45,004) reported no use of supplements; an additional 340 women had intakes < =1 μg/day.

Comparing participants (n = 33,425) to non-participants (n = 11,400) for the analyses of 18 month outcomes, participants were less likely to be nulliparous (55% vs. 60%) and they reported lower frequency of last menstrual period during the winter (25% vs. 30%). At 7 years, participants (n = 28,687 vs. 16,138 non-participants) were more likely to be of medium to higher socioeconomic status (58% vs. 53%), have a normal range prepregnancy BMI (70% vs. 65%), not to smoke during pregnancy (77% vs. 73%), and not exercise during pregnancy (60% vs. 64%). There were few differences in maternal age, birth weight, gestational age, and intake of micronutrients during pregnancy.

### Determinants of vitamin D intake

Table 1 shows the age-standardized distributions of covariates across quintiles of total vitamin D intake. Compared to women in the lower quintiles, women reporting higher consumption of total vitamin D were more likely to be nulliparous with a high level of proficiency and a normal range BMI. They tended to be non-smokers, be less exposed to second-hand smoke, and more likely to exercise. More women in the highest quintile used tanning beds during pregnancy. High vitamin D consumers had an overall healthier diet with higher fruit and vegetable intake, and higher consumption of folate, n-3 fatty acids, calcium, vitamins A, C, and E, selenium and zinc.

**Table 1:** Age-standardized covariates across quintiles of total vitamin D intake in 44,825 pregnant Danish women

	**Quintile of total maternal vitamin D in pregnancy**^**1**^				
	**1**	**2**	**3**	**4**	**5**
	**(n=8965)**	**(n=8965)**	**(n=8965)**	**(n=8965)**	**(n=8965)**
**Total Vitamin D (μg/day)**					
Median	5.5	8.2	11.7	13.0	16.5
5%-95%ile	1.8-7.1	7.3-9.4	9.9-12.3	12.4-14.0	14.2-27.4
**Vitamin D from supplement (μg/day)**					
Median	0	5.0	7.1	10.0	15.0
**Vitamin D from diet (μg/day)**					
Median	1.8	2.4	3.0	4.0	5.9
**Maternal age in years (mean±SD)**^**2**^					
<=20 (19.2±1.0)	2	1	2	2	1
20-25 (23.7±1.3)	19	16	20	20	16
25-30 (28.0±1.4)	44	45	46	45	46
30-35 (32.7±1.4)	28	30	25	26	29
>35 (37.6±1.7)	8	7	6	7	8
**Parity**					
Nulliparous	49	52	59	58	63
**Socioeconomic position**					
High level proficiencies	22	24	24	22	27
Medium level proficiencies	32	32	32	32	32
Skilled	28	27	27	28	25
Unskilled	13	12	12	13	11
Students	3	4	3	3	4
Unemployed	2	2	1	2	1
**Pre-pregnancy BMI in kg/m**^**2**^**(mean±SD)**					
<=18.5 (17.8±0.7)	4	4	4	5	4
18.6-24.9 (21.7±1.7)	67	69	68	67	70
25-29.9 (27.0±1.4)	20	20	20	20	18
>=30 (33.7±4.1)	8	8	8	8	7
**Breastfeeding duration**					
No breastfeeding	2	2	3	2	2
0-1 months	10	9	10	9	9
2-3 months	10	10	11	11	11
4-6 months	19	18	19	19	18
>=7 months	59	61	57	58	60
**Smoking in pregnancy**					
Nonsmoker	75	76	76	74	77
Occasional smoker	12	12	13	14	14
Current smoker	12	11	12	12	10
**Second-hand smoking**					
Yes	46	46	45	47	44
**Partner smoking**					
Yes	29	28	29	29	28
**Physical activity in min/week (mean±SD)**					
0 (0±0)	64	61	61	61	57
1-44 (29±7)	5	5	5	5	6
45-74 (57±7)	9	10	9	9	10
75-149 (105±19)	11	12	12	12	13
>=150(265±162)	11	12	12	12	14
**Outdoor activity in min/week (mean±SD)**					
0 (0±0)	83	83	83	83	82
1-59 (35±11)	3	2	2	2	2
60-119 (79±17)	5	6	5	5	6
>=120 (237±168)	9	9	10	9	10
**Maternal asthma**					
Yes	9	9	10	9	10
**Maternal allergy**					
Yes	32	32	32	32	33
**Paternal asthma**					
Yes	8	9	8	8	8
**Paternal allergy**					
Yes	24	25	25	24	26
**Season of LMP**					
Winter	27	26	26	25	27
Spring	25	23	25	26	24
Summer	21	24	23	24	23
Fall	27	27	26	25	26
**Total solarium use in pregnancy in weeks (mean±SD)**					
0 (0±0)	76	75	74	74	72
1-3 (1.5±0.7)	14	15	16	15	16
4-20 (9.7±4.8)	8	8	8	9	9
21-40 (30.3±4.0)	2	2	2	2	3
**Child sex (male)**	50	51	51	51	52
**Gestational weight gain (g/week)**	469(214)	468(211)	473(215)	474(213)	473(208)
**Gestational age (days)**	280(13)	280(13)	280(12)	280(13)	280(13)
**Birth weight (g)**	3,584(571)	3,598(571)	3,578(564)	3,572(567)	3,557(578)
**Maternal food and nutrient intake**					
Fruit intake (g/day)^3^	325(275)	319(255)	346(294)	332(265)	344(260)
Vegetables intake (g/day)^3^	125(98)	129(95)	130(98)	130(93)	148(106)
Energy (kJ/day)	10,073(2,683)	10,257(2,613)	9,919(2,637)	10,226(2,703)	9,727(2,663)
Alcohol (g/day)	1.6(2.3)	1.6(2.4)	1.5(2.1)	1.5(2.0)	1.6(2.3)
Total folate (μg/day)	466(188)	567(215)	680(162)	726(148)	758(224)
Total calcium (mg/day)	1,456(490)	1,541(486)	1,526(516)	1,582(519)	1,753(618)
Total EPA+DPA+DHA (mg/day)	48.3(318.3)	50.4(311.0)	65.0(359.9)	48.3(315.8)	75.4(396.8)
Total vitamin A (μg/day)	1,227(557)	1,659(469)	1,399(506)	1,351(439)	1,545(669)
Total vitamin E (mg/day)	16.4(34.8)	22.0(26.3)	19.6(29.1)	17.8(22.9)	23.8(40.0)
Total vitamin C (mg/day)	191(135)	228(134)	235(136)	229(125)	264(161)
Total selenium (μg/day)	62.9(25.2)	90.3(22.3)	80.3(21.2)	81.8(15.8)	92.5(28.5)
Total zinc (mg/day)	18.8(7.2)	25.9(5.3)	22.4(5.6)	21.9(4.9)	24.4(9.3)

Values are means(SD) or percentages and are standardized to the age distribution of the study population.

^1^% or means (SD).

^2^Value is not age adjusted.

^3^Including fruit/vegetable juices.

EPA: Eicosapentaenoic acid.

DPA: Docosapentaenoic acid.

DHA: Docosahexaenoic acid.

Similar relationships were found for dietary and supplemental vitamin D. Women who consumed more vitamin D from diet furthermore breastfed > =7 months and had their LMP in the winter months. Vitamin D supplement users showed no difference in socioeconomic position or smoking status (data not shown).

### Multivariable analyses

#### Ever doctor diagnosed asthma at 18 months

The prevalence of child asthma at 18 months was 17% (N = 5,635/33,425). We found no associations for either total vitamin D, vitamin D from supplements or diet (Tables 2 and 3; Additional file 1: Table S1). We found suggestion of an inverse relationship between dietary vitamin D and asthma diagnosis in the first 18 months of life, but this association disappeared after further adjustment for dietary covariates (Additional file 1: Table S1).

**Table 2:** **Associations**^** 1**^**between *total *** **vitamin D intake in mid-pregnancy and child asthma in the Danish National Birth Cohort**

**Quintile of intake**		**Cases/N**	**Asthma (18 months) N = 33,425/29,161**^**2**^**/28,421**^**3**^	**P for trend**^**4**^	**Cases/N**	**Current asthma (7 years - ISAAC) N = 28,687/25,025**^**2**^**/24,382**^**3**^	**P for trend**^**4**^	**Cases/N**	**Ever admitted asthma N = 28,767/25,095**^**2**^**/24,452**^**3**^	**P for trend**^**4**^	**Cases/N**	**Ever prescribed asthma N = 28,758/25,094**^**2**^**/25,452**^**3**^	**P for trend**^**4**^
			**RR (95% CI)**			**RR (95% CI)**			**RR (95% CI)**			**RR (95% CI)**	
Continuous	Crude	5,635/33,425 (17%)	1.00 (0.99, 1.00)	0.90	1,122/28,687 (4%)	1.00 (0.99, 1.01)	0.73	1,654/28,767 (6%)	1.00 (0.99, 1.01)	0.88	8,906/28,758 (31%)	1.00 (1.00, 1.01)	0.04
	Adjusted^2^		1.00 (1.00, 1.01)	0.59		1.00 (0.99, 1.01)	0.88		1.00 (0.99, 1.01)	0.45		1.01 (1.00, 1.01)	0.15
	Adjusted^3^		1.00 (0.99, 1.01)	0.91		0.99 (0.97, 1.00)	0.09		0.99 (0.97, 1.00)	0.08		1.00 (0.99, 1.00)	0.30
1	Crude	1,123/6,627 (17%)	1.00 (ref.)	0.54	219/5,728 (4%)	1.00 (ref.)	0.67	344/5,743 (6%)	1.00 (ref.)	0.92	1,724/5,741 (30%)	1.00 (ref.)	0.05
	Adjusted^2^			0.85			0.70			0.41			0.21
	Adjusted^3^			0.40			0.06			0.06			0.36
2	Crude	1,137/6,836 (17%)	0.98 (0.91, 1.06)		218/5,936 (4%)	0.96 (0.80, 1.15)		318/5,953 (5%)	0.89 (0.77, 1.03)		1,811/5,953 (30%)	1.01 (0.96, 1.07)	
	Adjusted^2^		1.01 (0.93, 1.09)			0.98 (0.81, 1.19)			0.92 (0.79, 1.08)			1.04 (0.98, 1.10)	
	Adjusted^3^		1.00 (0.91, 1.10)			0.84 (0.67, 1.05)			0.88 (0.73, 1.06)			0.99 (0.93, 1.06)	
3	Crude	1,170/6,725 (17%)	1.03 (0.95, 1.11)		231/5,710 (4%)	1.06 (0.88, 1.27)		341/5,725 (6%)	0.99 (0.86, 1.15)		1,798/5,721 (31%)	1.05 (0.99, 1.11)	
	Adjusted^2^		1.04 (0.96, 1.12)			1.02 (0.84, 1.24)			0.96 (0.82, 1.12)			1.04 (0.98, 1.10)	
	Adjusted^3^		1.00 (0.91, 1.11)			0.87 (0.69, 1.10)			0.90 (0.74, 1.09)			0.96 (0.90, 1.03)	
4	Crude	1,146/6,714 (17%)	1.01 (0.93, 1.09)		247/5,702 (4%)	1.13 (0.95, 1.35)		331/5,714 (6%)	0.97 (0.84, 1.12)		1,810/5,712 (32%)	1.06 (1.00, 1.11)	
	Adjusted^2^		1.01 (0.93, 1.09)			1.08 (0.90, 1.31)			0.92 (0.79, 1.08)			1.04 (0.98, 1.10)	
	Adjusted^3^		0.96 (0.86, 1.07)			0.91 (0.71, 1.17)			0.80 (0.65, 0.99)			0.95 (0.88, 1.03)	
5	Crude	1,059/6,523 (16%)	0.96 (0.89, 1.03)		207/5,611 (4%)	0.96 (0.80, 1.16)		320/5,632 (6%)	0.95 (0.82, 1.10)		1,763/5,631 (31%)	1.04 (0.99, 1.10)	
	Adjusted^2^		0.99 (0.91, 1.07)			0.91 (0.75, 1.11)			0.92 (0.79, 1.08)			1.04 (0.98, 1.11)	
	Adjusted^3^		0.96 (0.86, 1.07)			0.74 (0.56, 0.96)			0.80 (0.64, 1.00)			0.97 (0.89, 1.05)	

^1^Analyzed using log-binomial models.

^2^Adjusted for maternal age, socio-economic status, parity, prepregnancy BMI, smoking during pregnancy, partner’s smoking during pregnancy, solarium use during pregnancy, breastfeeding duration, child sex, maternal history of asthma, maternal history of allergies, paternal history of asthma, paternal history of allergies, season of last menstrual period, and energy (in quintiles).

^3^Adjusted as in ^2^ plus fruit and vegetable intake, alcohol consumption, and intake of total EPA + DPA + DHA, vitamins A, C, and E, folate, calcium, selenium, and zinc (all in quintiles).

^4^Median values for each quintile entered as a continuous variable into the model. For continuous exposure the P-value reflects the P-value of the effect estimate.

ISAAC: International Study of Asthma and Allergies in Childhood.

EPA: Eicosapentaenoic acid.

DPA: Docosapentaenoic acid.

DHA: Docosahexaenoic acid

**Table 3:** **Associations**^**1**^**between vitamin D intake from** ***supplements*** **in mid-pregnancy and child asthma in the Danish National Birth Cohort**

**Quintile of intake**		**Cases/N**	**Asthma (18 months)N = 33,425/29,161**^**2**^**/28,421**^**3**^	**P for trend**^**4**^	**Cases/N**	**Current asthma (7 years - ISAAC)N = 28,686/25,024**^**2**^**/24,382**^**3**^	**P for trend**^**4**^	**Cases/N**	**Ever admitted asthmaN = 28,766/25,094**^**2**^**/24,452**^**3**^	**P for trend**^**4**^	**Cases/N**	**Ever prescribed asthmaN = 28,757/25,093**^**2**^**/24,452**^**3**^	**P for trend**^**4**^
			**RR (95% CI)**			**RR (95% CI)**			**RR (95% CI)**			**RR (95% CI)**	
Continuous	Crude	5,635/33,425 (17%)	1.01 (1.00, 1.01)	0.04	1,122/28,686 (4%)	1.01 (0.99, 1.02)	0.40	1,654/28,766 (6%)	1.00 (0.99, 1.01)	0.52	8,905/28,757 (31%)	1.01 (1.01, 1.01)	<0.0001
	Adjusted^2^		1.00 (1.00, 1.01)	0.14		1.00 (0.99, 1.01)	0.99		1.00 (0.99, 1.01)	0.52		1.01 (1.00, 1.01)	0.01
	Adjusted^3^		1.00 (0.99, 1.01)	0.85		0.98 (0.97, 1.00)	0.10		0.99 (0.97, 1.00)	0.07		1.00 (0.99, 1.00)	0.65
1	Crude	835/5,071 (16%)	1.00 (ref.)	0.06	158/4,362 (4%)	1.00 (ref.)	0.18	251/4,375 (6%)	1.00 (ref.)	0.51	1,216/4,374 (28%)	1.00 (ref.)	<0.0001
	Adjusted^2^			0.38			0.84			0.36			0.005
	Adjusted^3^			0.55			0.14			0.02			0.79
2	Crude	1,675/10,142 (17%)	1.00 (0.93, 1.08)		348/8,849 (4%)	1.09 (0.90, 1.31)		498/8,873 (6%)	0.98 (0.84, 1.13)		2,736/8,872 (31%)	1.11 (1.05, 1.17)	
	Adjusted^2^		1.01 (0.94, 1.10)			1.07 (0.88, 1.30)			0.98 (0.84, 1.14)			1.12 (1.05, 1.19)	
	Adjusted^3^		1.03 (0.91, 1.17)			1.05 (0.78, 1.41)			1.00 (0.78, 1.27)			1.11 (1.01, 1.22)	
3	Crude	266/1,761 (15%)	0.92 (0.81, 1.04)		49/1,589 (3%)	0.85 (0.62, 1.17)		80/1,590 (5%)	0.88 (0.69, 1.12)		440/1,589 (28%)	1.00 (0.91, 1.09)	
	Adjusted^2^		0.95 (0.83, 1.08)			0.78 (0.55, 1.10)			0.84 (0.64, 1.09)			1.02 (0.92, 1.12)	
	Adjusted^3^		0.91 (0.78, 1.05)			0.62 (0.42, 0.90)			0.71 (0.53, 0.95)			0.96 (0.86, 1.07)	
4	Crude	2,443/14,064 (17%)	1.05 (0.98, 1.13)		481/11,828 (4%)	1.12 (0.94, 1.34)		705/11,866 (6%)	1.04 (0.90, 1.19)		3,842/11,860 (32%)	1.16 (1.10, 1.23)	
	Adjusted^2^		1.02 (0.95, 1.11)			1.06 (0.88, 1.27)			0.97 (0.83, 1.12)			1.12 (1.05, 1.18)	
	Adjusted^3^		0.99 (0.87, 1.11)			0.97 (0.73, 1.29)			0.84 (0.67, 1.07)			1.05 (0.96, 1.15)	
5	Crude	417/2,387 (17%)	1.06 (0.95, 1.18)		86/2,058 (4%)	1.15 (0.89, 1.49)		120/2,062 (6%)	1.01 (0.82, 1.25)		671/2,062 (33%)	1.17 (1.08, 1.27)	
	Adjusted^2^		1.05 (0.94, 1.17)			1.01 (0.77, 1.34)			0.88 (0.70, 1.11)			1.11 (1.02, 1.21)	
	Adjusted^3^		0.99 (0.86, 1.14)			0.81 (0.57, 1.15)			0.77 (0.58, 1.03)			1.03 (0.93, 1.15)	

^1^Analyzed using log-binomial models.

^2^Adjusted for maternal age, socio-economic status, parity, prepregnancy BMI, smoking during pregnancy, partner’s smoking during pregnancy, solarium use during pregnancy, breastfeeding duration, child sex, maternal history of asthma, maternal history of allergies, paternal history of asthma, and paternal history of allergies, season of last menstrual period, and energy (in quintiles).

^3^Adjusted as in ^2^ plus fruit and vegetable intake, alcohol consumption, and intake of total EPA + DPA + DHA, vitamins A, C, and E, folate, calcium, selenium, and zinc (all in quintiles).

^4^Median values for each quintile entered as a continuous variable into the model. For continuous exposure the P-value reflects the P-value of the effect estimate.

ISAAC: International Study of Asthma and Allergies in Childhood.

EPA: Eicosapentaenoic acid.

DPA: Docosapentaenoic acid.

DHA: Docosahexaenoic acid.

#### Current and ever asthma at 7 years

At age 7 years 6% (N = 1,654/28,767) of children were classified with ever admitted asthma and 31% (N = 8,906/28,758) with ever prescribed asthma. The prevalence of current asthma at age 7 was 4% (N = 1,122/28,687). After adjustment for sociodemographic and dietary covariates, we found an inverse, though modest, association for total vitamin D intake with both current asthma at age 7 (continuous: 0.99, 95% CI: 0.97, 1.00, P = 0.09; Q5 vs. Q1: 0.74, 95% CI: 0.56, 0.96) and ever admitted asthma (continuous: 0.99, 95% CI: 0.97, 1.00, P = 0.08; Q5 vs. Q1: 0.80, 95% CI: 0.64, 1.00) (Table 2). We found null relationships for vitamin D from supplements and diet; though a tendency towards an inverse association was present for continuous vitamin D from supplements with both current asthma and ever admitted asthma (Table 3). We found no association for any of the vitamin D exposures with ever prescribed asthma (Tables 2 and 3; Additional file 1: Table S1).

Since 1995 the DNPR has contained information on whether patients are diagnosed as inpatients, outpatients, or in an emergency department (ED). There were too few ED cases to allow models to converge, but for inpatient cases we found an inverse relation between total vitamin D consumption and admitted asthma (Q5 (171/5,304) vs. Q1 (190/5,387): 0.73, 95% CI: 0.54, 1.00, P = 0.04).

#### Ever allergic rhinitis at 7 years

The prevalence of child ever allergic rhinitis was 5% (N = 1,420/28,545). No associations were found for either total vitamin D intake, or vitamin D from supplements or diet (Table 4).

**Table 4:** **Associations**^**1**^**between vitamin D intake in mid-pregnancy and self-reported child allergic rhinitis in the Danish National Birth Cohort**

**Quintile of intake**		**Cases/N**	**Allergic rhinitis by total vitamin DN = 28,545/24,902**^**2**^**/24,261**^**3**^	**P for trend**^**4**^	**Cases/N**	**Allergic rhinitis by vitamin D from supplementsN = 28,544/24,901**^**2**^**/24,261**^**3**^	**P for trend**^**4**^	**Cases/N**	**Allergic rhinitis by vitamin D from dietN = 28,545/24,902**^**2**^**/24,261**^**3**^	**P for trend**^**4**^
			**RR (95% CI)**			**RR (95% CI)**			**R (95% CI)**	
Continuous	Crude	1,420/28,545 (5%)	1.00 (1.00, 1.02)	0.33	1,420/28,544 (5%)	1.00 (0.99, 1.01)	0.51	1,420/28,545 (5%)	1.01 (0.99, 1.04)	0.31
	Adjusted^2^		1.00 (0.99, 1.01)	0.53		0.99 (0.98, 1.01)	0.38		1.01 (0.98, 1.03)	0.62
	Adjusted^3^		1.00 (0.99, 1.01)	0.97		1.00 (0.98, 1.01)	0.81		1.01 (0.97, 1.04)	0.65
1	Crude	263/5,700 (5%)	1.00 (ref.)	0.10	190/4,344 (4%)	1.00 (ref.)	0.28	296/5,646 (5%)	1.00 (ref.)	0.63
	Adjusted^2^			0.84			0.56			0.87
	Adjusted^3^			0.62			0.94			0.67
2	Crude	292/5,903 (5%)	1.07 (0.91, 1.26)		457/8,801 (5%)	1.19 (1.01, 1.40)		263/5,673 (5%)	0.88 (0.75, 1.04)	
	Adjusted^2^		1.05 (0.89, 1.25)			1.12 (0.94, 1.33)			0.91 (0.76, 1.07)	
	Adjusted^3^		1.10 (0.90, 1.34)			1.23 (0.95, 1.60)			0.91 (0.76, 1.08)	
3	Crude	284/5,683 (5%)	1.08 (0.92, 1.28)		65/1,580 (4%)	0.94 (0.71, 1.24)		265/5,742 (5%)	0.88 (0.75, 1.03)	
	Adjusted^2^		1.01 (0.85, 1.19)			0.96 (0.72, 1.27)			0.90 (0.76, 1.07)	
	Adjusted^3^		1.14 (0.92, 1.41)			1.07 (0.78, 1.47)			0.88 (0.73, 1.08)	
4	Crude	284/5,674 (5%)	1.08 (0.92, 1.28)		614/11,773 (5%)	1.19 (1.02, 1.40)		311/5,810 (5%)	1.02 (0.87, 1.19)	
	Adjusted^2^		1.02 (0.86, 1.21)			1.06 (0.90, 1.26)			1.05 (0.89, 1.23)	
	Adjusted^3^		1.11 (0.89, 1.40)			1.23 (0.95, 1.58)			1.04 (0.85, 1.28)	
5	Crude	297/5,585 (5%)	1.15 (0.98, 1.35)		94/2,046 (5%)	1.05 (0.83, 1.34)		286/5,674 (5%)	0.96 (0.82, 1.13)	
	Adjusted^2^		1.00 (0.84, 1.19)			0.90 (0.70, 1.16)			0.92 (0.78, 1.09)	
	Adjusted^3^		1.08 (0.85, 1.37)			1.01 (0.74, 1.39)			0.89 (0.71, 1.13)	

^1^Analyzed using log-binomial models.

^2^Adjusted for maternal age, socio-economic status, parity, prepregnancy BMI, smoking during pregnancy, partner’s smoking during pregnancy, solarium use during pregnancy, breastfeeding duration, child sex, maternal history of asthma, maternal history of allergies, paternal history of asthma, and paternal history of allergies, season of last menstrual period, and energy (in quintiles).

^3^Adjusted as in ^2^ plus fruit and vegetable intake, alcohol consumption, and intake of total EPA + DPA + DHA, vitamins A, C, and E, folate, calcium, selenium, and zinc (all in quintiles).

^4^Median values for each quintile entered as a continuous variable into the model. For continuous exposure the P-value reflects the P-value of the effect estimate.

ISAAC: International Study of Asthma and Allergies in Childhood.

EPA: Eicosapentaenoic acid.

DPA: Docosapentaenoic acid.

DHA: Docosahexaenoic acid.

#### Sensitivity analyses

We further examined any wheeze and recurrent wheeze (>3 episodes vs. <=3 episodes or no wheeze) at 18 months and found no associations for any exposure after adjusting for covariates (data not shown).

Categorizing the total vitamin D intake in 5 μg/day increments (from zero to > =25 μg/day) or 2.5 μg/day (from zero to > =15 μg/day) did not show any association, except for asthma at 18 months which showed an inverse association for total vitamin D > =20 μg/day vs. none (0.76, 95% CI: 0.59, 0.96, P = 0.02). Examining vitamin D intake by dichotomizing it according to US (15 μg/day) and Nordic (10 μg/day) recommendations, we found no associations with any of the outcomes (data not shown).

Respiratory symptoms in early childhood may involve pathologies and etiologies that are different from clinical asthma [45]. We therefore excluded the first 3 years of life from the registry-based outcomes. The number of admitted cases more than halved, and the number of prescription-related asthma cases was reduced by close to 80%. This reduced the significant associations to null, altering the effect estimates and widening the confidence intervals. The association between total vitamin D and ever prescribed asthma was borderline significant (Q5 vs. Q1: 0.82, 95% CI: 0.67, 1.00, P = 0.05).

We found no interactions by parental history of asthma and allergy, or by maternal vitamin A intake. Stratifying on maternal prepregnancy BMI, we found that the associations for maternal total vitamin D intake with child current asthma at age 7 (Q5 vs. Q1: 0.67, 95% CI: 0.49, 0.92, P = 0.01) and ever admitted asthma (Q5 vs. Q1: 0.74, 95% CI: 0.56, 0.96, P = 0.03) were stronger for women with a BMI between 18.6 and 24.9 kg/m^2^ compared to women with a BMI > =25 kg/m^2^ where the results were null. Women in the < =18.5 kg/m^2^ group were too few to assess any meaningful effect modification.

## Discussion

In this large, longitudinal study of over 40,000 mother-child dyads with detailed dietary and supplement data in pregnancy, we found weak inverse associations for maternal total vitamin D intake with ever admitted and current child asthma at age 7 only. This association appeared to be accounted for by supplemental vitamin D as two-thirds of maternal intake came from supplements and trends towards similar associations were found for supplemental vitamin D. We found no associations for asthma at 18 months, ever prescribed asthma, or allergic rhinitis.

The total vitamin D intake in our study population had a mean of 11.2 μg/day with a 5^th^-95^th^% of 3.0-19.4 μg/day (or 440 IU/day; 5^th^-95^th^%: 120-776 IU/day), with 304 IU/day coming from supplements. This is higher than what has been found in Finnish (204 IU/day, no range given) [28], UK (137 IU/day, 5^th^-95^th^%: 67-445 IU/day) [20], and Japanese (248 IU/day, no range given) [21] populations, which all had low supplement use (5-30% vs. 68% in the DNBC) [46]; but lower than in a US cohort (548 IU/day; 319 IU/day from supplements; range 60–1,145 IU/day) [19]. Differences in intake range from supplements vs. diet between our and prior studies could explain why our significant results were limited to vitamin D from supplements. Dietary recommendations for vitamin D during pregnancy are subject to debate and uncertainty exists regarding optimal levels needed to attain potential benefits of vitamin D [47]. In Denmark, 25(OH)D levels in healthy, non-pregnant volunteers have ranged from 27 to 91 nmol/L [10,48-53] with deficiency varying from 9% to 30% [10,48,54]. Measuring 25(OH)D levels in 1,494 women in the DNBC, we found mean(SD) 25(OH)D levels of 56.7(24.6) nmol/L; 42.3% of women were classified as vitamin D deficient (<50 nmol/L) [11]. Some have argued that circulating 25(OH)D levels in excess of 100 nmol/L are necessary to optimize production of 1,25(OH)_2_D [55] and that dietary intake of as much as 4,000 IU/day may be needed to reach those levels for certain deficient populations [47,56]. The Nordic Council of Ministers currently recommends a daily intake of 10 μg/day (400 IU/day) of vitamin D for all pregnant women [39]. Our results did not indicate protection against child asthma at this dosage. Rather, benefits for child asthma appear to be derived at higher intake levels found in the two upper quintiles. It is possible that more protective effects could have been found at even higher intake levels, but the range of intake in our data did not allow us to examine this. Furthermore, we found that the results were limited to women with a prepregnancy BMI between 18.6 and 24.9 kg/m^2^. Increased adiposity may decrease maternal bioavailability of vitamin D [57] and consequently reduce fetal exposure to vitamin D. This finding needs to be confirmed in future studies.

There have been few studies examining the association between child asthma and vitamin D from dietary sources and supplement use [19-21,27,28]. These studies have all found inverse associations specific to diet, but were inconsistent across child asthma and allergic disease outcomes. Majority of studies found that higher intake of dietary vitamin D protected against wheeze up to 5 years [19-21]; our results suggested only weak, non-significant associations of dietary vitamin D with wheeze and recurrent wheeze that disappeared after adjusting for dietary confounders. One study also showed an inverse association for vitamin D from supplements and recurrent wheeze at age 3 [19]. Maternal dietary vitamin D also seems to reduce the child’s risk of eczema [21], bronchodilator response [20], allergic rhinitis [28] and asthma [28]; we similarly found an inverse association for asthma by self-report and hospital admission at age 7. We did not find any evidence of an association between maternal vitamin D intake and offspring allergic rhinitis or other asthma outcomes.

Despite a growing number of mechanistic studies, little is still understood about vitamin D and allergic disease development. A primary mechanism in explaining the potential relationship between vitamin D exposure and allergic disease has involved Treg cells, a newly uncovered set of immune cells with suppressive and regulatory functions [58]. Animal and *in vitro* studies have suggested that Treg cells may play a role in the suppression of the Th2 response often found exaggerated in patients with allergic disease [59,60], and that this suppression mechanism may be flawed in allergic patients [61,62]. Vitamin D may influence Treg cells through increased cell production [14,63] and by stimulating IL-10 production, an anti-inflammatory and immunosuppressive cytokine that is also important for Treg activation [64]. Mechanisms have also included modification of antigen-presenting cell function [65], inhibition of IgE production [66], and protection against airway remodeling [67] and inflammation [68]. Other studies seem to suggest that vitamin D may enhance the Th2 response [69,70] or that dual responses may exist [71]. Pichler et al. [72] demonstrated that vitamin D both reduced IL-12–generated interferon -γ production and suppressed IL-4 and IL-4 induced expression of IL-13 in naive Th cells and cytotoxic T cells. These seemingly contradictory findings may point to the importance of timing of exposure in prenatal vs. mature immune cells. While our data did not enable us to conclude anything about timing of exposure, examining exposure at different time points during pregnancy may be important in clarifying these differences.

This study adds to the evidence on vitamin D intake in pregnancy and child allergic disease and further research on early respiratory outcomes. Strengths of our study include following a large, prospective cohort with comprehensive data on vitamin D from both diet and supplements. We were also able to include an extensive outcome assessment using data from questionnaires and two population-based disease registries, a unique strength in our setting. We included outcomes in both early and later childhood, taking into consideration potentially different etiologies of similar symptom manifestations across childhood. Early wheeze and asthma diagnosis made in the first years of life may primarily include transient or viral wheeze, which would explain the higher prevalence of asthma at 18 months [45]. On the other hand, asthma development peaks at around 5–6 years; hence, later diagnosis and clinical diagnoses in registries may capture more clinical asthma. For our later outcomes we used questions validated by ISAAC and a patient registry that has demonstrated good validity in previous studies [73]. We found a slightly stronger association for inpatient diagnoses, suggesting potential importance of disease severity. The significant findings in this analysis appeared to be influenced by adjustment for dietary variables. Upon further examination we found that total zinc intake was responsible for the greatest change in effect estimates for ever admitted asthma. No single nutrient appeared to drive the changes for current asthma at age 7. Maternal total zinc intake was inversely related to total vitamin D consumption and to child asthma, independent of other dietary variables. This suggests that zinc may be a positive confounder and explain the downward shift in effect estimates after adjustment. We believe that the adjustment for both sociodemographic and dietary covariates minimized residual confounding.

Our study had some potential limitations. Maternal dietary exposure was self-reported and therefore prone to misclassification; yet, we expect any misclassification to have been non-differential and underestimate the strength of observed associations. This together with the low levels and low variability in intake, especially for diet, may have limited our ability to capture significant associations. We used maternal dietary exposure during one time point in pregnancy. Other time intervals may be important and need to be examined more closely in order to establish the period of etiological relevance. Although we did not have access to serum levels of vitamin D, we considered numerous determinants of vitamin D status in our models, including leisure time activity, outdoor occupation, tanning beds and vacationing in sun-exposed countries. While serum levels give a measure of vitamin D exposure from diet and sunlight, supplementation is more pertinent in making recommendations to pregnant women. We cannot exclude that correlations with other nutrients may be responsible for the observed associations; however, Spearman correlations coefficients for nutrients included as covariates, such as total vitamin E and C, zinc and selenium intake, were all below 0.25 (data not shown).

In order to capture potentially different asthmatic phenotypes we included asthma outcomes from different sources, and we were not able to demonstrate an effect of the exposure on two of the asthma outcomes. Asthma at 18 months may have largely consisted of transient wheeze, while ever prescribed asthma may have included cases ranging from suspect asthma to more severe disease in need of medication [74]. Consequently, a lower specificity for the latter and a subsequent attenuating of the effect measures may account for the failure to detect an association. The exclusion of potential transient wheeze in the first three years of life, considerably reducing the number of cases, strengthened the association for ever prescribed asthma, but it did not reach statistical significance. Finally, attrition is a concern in longitudinal studies in regards to potential for selection bias. However, upon examining characteristics between participants and non-participants we found few minor differences, suggesting limited bias.

## Conclusions

In this large, prospective study of maternal dietary and supplement vitamin D intake in mid-pregnancy and child asthma, we found weak inverse associations for total vitamin D with ever admitted asthma and current asthma at 7 years, but not with outcomes at 18 months or ever allergic rhinitis at 7 years. We cannot exclude that these associations may have been due to residual confounding. Although the vitamin D intake in our population was in accordance with the current recommendations for intake during pregnancy, the inverse association was not seen at recommended dosage level. Two clinical trials of maternal vitamin D supplementation and child asthma (NCT00920621 and NCT00856947) are currently underway. While randomized clinical trials are considered to be the gold standard, observational studies may be more appropriate for answering questions on nutrient dosage. As noted in a recent commentary, trials are limited by the nutrient range that can be examined and the baseline nutrient level that can be included due to both feasibility and ethical issues, and follow-up of diseases developing years later may not be possible in most settings [75].

Future studies should delineate the importance of exposure timing as well as relevant dosage level in pregnancy as this will have clinical relevance in making recommendations to pregnant women.

## Abbreviations

DNBC: Danish National Birth Cohort; FFQ: Food frequency questionnaire; LMP: Last menstrual period; DNPR: Danish National Patient Registry; RMPS: Register of Medicinal Product Statistics; RR: Risk ratio; ED: Emergency department.

## Competing interest

The author(s) declare that they have no competing interests.

## Authors’ contributions

The authors’ responsibilities were as follows – EM and SFO: study concept and design; EM: prepared the data and conducted the statistical analyses; EM and SH: drafted the manuscript; EM, SH, CBJ, ALTL, MS, SFO: contributed critical advice and revisions of the manuscript; SFO: funding; EM and SFO: acquisition of data and responsibility for the entire contents of the manuscript. All authors had full access to study data. All authors read and approved the final manuscript.

## Pre-publication history

The pre-publication history for this paper can be accessed here:

http://www.biomedcentral.com/1471-2393/13/199/prepub

## Supplementary Material

Additional file 1: Table S1 Associations between vitamin D intake from diet in mid-pregnancy and child asthma in the Danish National Birth Cohort. Click here for file
