# Supplementary material for: Vitamin D intake in mid-pregnancy and child allergic disease – a prospective study in 44,825 Danish mother-child pairs
Source: BMC Pregnancy Childbirth. 2013 Oct 31;13:199. doi: 10.1186/1471-2393-13-199 (PMC3871013; doi:10.1186/1471-2393-13-199)
Supplement: Additional file 1: Table S1 — Associations between vitamin D intake from diet in mid-pregnancy and child asthma in the Danish National Birth Cohort. [file 1471-2393-13-199-S1.docx]

Additional file 1: Table S1. Associations^1^ between vitamin D intake from *diet* in mid-pregnancy and child asthma in the Danish National Birth Cohort

| **Quintile of intake** |  | **Cases/N** | **Asthma**  **(18 months)**  N=33,425/  29,161^2^/  28,421^3^  RR (95% CI) | **P for trend**^4^ | **Cases/N** | **Current asthma**  **(7 years - ISAAC)**  N=28,687/  25,025^2^/  24,382^3^  RR (95% CI) | **P for trend**^4^ | **Cases/N** | **Ever admitted asthma**  N=28,767/  25,095^2^/  24,452^3^  RR (95% CI) | **P for trend**^4^ | **Cases/N** | **Ever prescribed asthma**  N=28,758/  25,094^2^/  24,452^3^  RR (95% CI) | **P for trend**^4^ |
| --- | --- | --- | --- | --- | --- | --- | --- | --- | --- | --- | --- | --- | --- |
| Continuous | Crude  Adjusted^2^  Adjusted^3^ | 5,635/33,425 (17%) | 0.97 (0.95, 0.98)  0.99 (0.97, 1.00)  0.99 (0.97, 1.01) | <0.0001  0.04  0.43 | 1,122/28,687 (4%) | 0.99 (0.96, 1.01)  1.00 (0.97, 1.03)  0.99 (0.95, 1.03) | 0.32  0.79  0.68 | 1,654/28,767 (6%) | 0.98 (0.95, 1.00)  1.00 (0.97, 1.02)  1.00 (0.96, 1.03) | 0.08  0.72  0.82 | 8,906/28,758 (31%) | 0.97 (0.96, 0.98)  0.99 (0.98, 1.00)  0.99 (0.98, 1.00) | <0.0001  0.02  0.11 |
|  |  |  |  |  |  |  |  |  |  |  |  |  |  |
| 1 | Crude  Adjusted^2^  Adjusted^3^ | 1,181/6,635 (18%) | 1.00 (ref.) | <0.0001  0.11  0.86 | 245/5,682 (4%) | 1.00 (ref.) | 0.30  0.95  0.99 | 377/5,699 (7%) | 1.00 (ref.) | 0.06  0.72  0.92 | 1,867/5,695 (33%) | 1.00 (ref.) | <0.0001  0.06  0.40 |
| 2 | Crude  Adjusted^2^  Adjusted^3^ | 1,197/6,782 (18%) | 0.99 (0.92, 1.07)  1.02 (0.94, 1.10)  1.01 (0.93, 1.10) |  | 201/5,702 (4%) | 0.82 (0.68, 0.98)  0.84 (0.69, 1.02)  0.83 (0.68, 1.02) |  | 324/5,712 (7%) | 0.86 (0.74, 0.99)  0.88 (0.75, 1.03)  0.86 (0.73, 1.02) |  | 1,831/5,711 (32%) | 0.98 (0.93, 1.03)  0.99 (0.94, 1.05)  0.99 (0.93, 1.09) |  |
| 3 | Crude  Adjusted^2^  Adjusted^3^ | 1,144/6,801  (17%) | 0.95 (0.88, 1.02)  0.96 (0.88, 1.03)  0.96 (0.87, 1.05) |  | 246/5,777 (4%) | 0.99 (0.83, 1.17)  1.03 (0.85, 1.23)  0.98 (0.79, 1.22) |  | 320/5,792 (6%) | 0.84 (0.72, 0.96)  0.88 (0.75, 1.02)  0.86 (0.72, 1.03) |  | 1,803/5,791 (31%) | 0.95 (0.90, 1.00)  0.98 (0.92, 1.03)  0.98 (0.92, 1.04) |  |
| 4 | Crude  Adjusted^2^  Adjusted^3^ | 1,139/6,685 (17%) | 0.96 (0.89, 1.03)  1.03 (0.95, 1.11)  1.04 (0.95, 1.15) |  | 215/5,835 (4%) | 0.85 (0.71, 1.02)  0.91 (0.75, 1.10)  0.87 (0.68, 1.10) |  | 315/5,851 (5%) | 0.81 (0.70, 0.94)  0.89 (0.77, 1.05)  0.87 (0.72, 1.07) |  | 1,793/5,849 (31%) | 0.94 (0.89, 0.99)  1.00 (0.94, 1.05)  1.01 (0.94, 1.08) |  |
| 5 | Crude Adjusted^2^  Adjusted^3^ | 974/6,522 (15%) | 0.84 (0.78, 0.91)  0.93 (0.86, 1.01)  0.98 (0.87, 1.10) |  | 215/5,691 (4%) | 0.88 (0.73, 1.05)  0.97 (0.80, 1.17)  0.95 (0.73, 1.25) |  | 318/5,713 (6%) | 0.84 (0.73, 0.97)  0.93 (0.80, 1.09)  0.93 (0.74, 1.16) |  | 1,612/5,712 (28%) | 0.86 (0.81, 0.91) 0.94 (0.89, 1.00)  0.96 (0.88, 1.04) |  |

^1^Analyzed using log-binomial models.

^2^Adjusted for maternal age, socio-economic status, parity, prepregnancy BMI, smoking during pregnancy, partner’s smoking during pregnancy, solarium use during pregnancy, breastfeeding duration, child sex, maternal history of asthma, maternal history of allergies, paternal history of asthma, and paternal history of allergies, season of last menstrual period, and energy (in quintiles).

^3^Adjusted as in ^2^ plus fruit and vegetable intake, alcohol consumption, and intake of total EPA + DPA + DHA, vitamins A, C, and E, folate, calcium, selenium, and zinc (all in quintiles).

^4^Median values for each quintile entered as a continuous variable into the model. For continuous exposure the P-value reflects the P-value of the effect estimate.

ISAAC: International Study of Asthma and Allergies in Childhood

EPA: eicosapentaenoic acid

DPA: docosapentaenoic acid

DHA: docosahexaenoic acid
